# Delivery of Mesenchymal Stem Cell in Dialdehyde Methylcellulose-Succinyl-Chitosan Hydrogel Promotes Chondrogenesis in a Porcine Model

**DOI:** 10.3390/polym14071474

**Published:** 2022-04-05

**Authors:** Yu-Chun Chen, Hsiu-Jung Liao, Yuan-Ming Hsu, Yi-Shan Shen, Chih-Hung Chang

**Affiliations:** 1 Department of Chemical Engineering, National United University, Miaoli 360302, Taiwan; f94548002@gmail.com; 2 Department of Orthopedic Surgery, Far Eastern Memorial Hospital, New Taipei City 220216, Taiwan; liaohsiujung@gmail.com (H.-J.L.); femhortho@gmail.com (Y.-M.H.); katrina60943@gmail.com (Y.-S.S.); 3 Department of Biomedical Engineering, National Taiwan University, Taipei 10617, Taiwan; 4 Graduate School of Biotechnology and Bioengineering, Yuan Ze University, Taoyuan City 320315, Taiwan

**Keywords:** dialdehyde methylcellulose, succinyl-chitosan, hydrogel, chondrogenesis, mesenchymal stem cell

## Abstract

Due to the limitation in the current treatment modalities, such as secondary surgery in ACI and fibrocartilage formation in microfracture surgery, various scaffolds or hydrogels have been developed for cartilage regeneration. In the present study, we used sodium periodate to oxidize methylcellulose and formed dialdehyde methylcellulose (DAC) after dialysis and freeze-drying process, DAC was further mixed with succinyl-chitosan (SUC) to form an DAC-SUC in situ forming hydrogel. The hydrogel is a stiffness, elastic-like and porous hydrogel according to the observation of SEM and rheological analysis. DAC-SUC13 hydrogel possess well cell-compatibility as well as biodegradability. Most bone marrow mesenchymal stem cells (BM-pMSCs) were alive in the hydrogel and possess chondrogenesis potential. According to the results of animal study, we found DAC-SUC13 hydrogel can function as a stem cell carrier to promote glycosaminoglycans and type II collagen synthesis in the osteochondral defects of porcine knee. These findings suggested that DAC-SUC13 hydrogel combined with stem cell is a potential treatment for cartilage defects repair in the future.

## 1. Introduction

Aging, sport injury, inflammation and genetic predisposition may lead to cartilage degraded and are strongly associated with higher incidences of degenerative osteoarthritis and cartilage lesions [1]. The capacity of intrinsic healing in cartilages is limited due to the lack of blood vessels and lower metabolic activities of chondrocytes [2]. Clinical treatments for cartilage lesions in non-surgical or surgical management, including hormone therapy, cytokine therapy, and microfracture have some limitations, such as severe systemic side effects, fibrocartilage formation and limited cartilage mass [3]. Thus far, there is no effective therapeutic strategy to stimulate articular cartilage regeneration [4,5].

Autologous chondrocyte implantation (ACI) is another well-known surgical procedure for cartilage defect repair, doctors take out patients’ articular cartilage from unloading site, and the cells (chondrocyte) within tissue be collected, proliferated in a specific laboratory for cell number increase [6,7]. When the cell number is enough, patients need to return to hospital for autologous chondrocyte implantation. The cartilage defect site is then covered with periosteum and delivered the proliferated chondrocyte with small gauge needle [8]. However, such kinds of therapy possess some limitations such as secondary surgery and implanted chondrocyte loss. Thus, for steadily transplanting the autologous cells to the injured cartilages, we developed an in situ forming methylcellulose/chitosan mixed hydrogel as a vehicle for cell encapsulation.

Cellulose is a linear homopolymer of D-glucose units with β (1→4) linkage, and methyl cellulose is the simplest cellulose derivative. The methyl groups (–CH_3_) may substitute the hydroxyls at C2, C3 or C6 positions to improve its water solubility [9]. It has been used in medical related studies, such as constipation treatment and artificial tears [10]. Recently, some research extended its use to cartilage regeneration due to its high strength in the wet state, biocompatibility as well as relatively simple, cost-efficient production [11,12,13]. Various types of cellulose-based scaffold were developed for chondrogenesis enhancement, such as membrane [14], double network [15], oxidation [16] and electrospinning [17]. Chitosan is composed of glucosamine and *N*-acetylglucosamine and has a similar structure to some articular cartilage components [18,19]. It has limited solubility in physiological solvents because of the strong intermolecular hydrogen bonding. To improve its solubility, we introduced succinyl groups at the N-position of the glucosamine units to synthesize *N*-succinyl-chitosan (SUC). In the study, we created dialdehyde functional group on methyl cellulose (DAC) at C2 and C3 position by oxidation process, mixed with amine group rich molecular-*N*-succinyl-chitosan (SUC) to form an in situ forming DAC-SUC hydrogel without other crosslinking reagents.

Mesenchymal stem cells (MSCs) can be taken from a donor’s bone marrow, they have a high chondrogenic potential and been most extensively explored for cell-based therapy for cartilage repair [20,21,22]. To improve the secondary surgery and donor site morbidity limitation of ACI, we used bone marrow mesenchymal stem cells (BM-MSCs) as a cell source for cartilage repair, bone marrow can be isolated before the surgery for cell proliferation until cell number enough [23]. Patients only need to conduct the surgery once for in situ forming MSC/methylcellulose/chitosan hydrogel injection.

To evaluate the repair effect in vivo, we use porcine as an animal model. It is a promising donor in cartilage research due to the joint size, cartilage thickness, and limited capability for the endogenous repair of osteochondral defects (OCD) [24]. These characteristics are more similar to humans, and porcine is considered as an appropriate large-animal model for preclinical studies. We created osteochondral defect in medial condyles of both porcine’s knee joints and used BM-pMSCs as a cell source to assess the cartilage repair effect of BM-pMSCs embedded in DAC-SUC hydrogel.

We hypothesized that a combination of BM-pMSCs and hydrogel may synergistically enhance the efficiency of cartilage matrix synthesis and promote cartilage repair in an osteochondral defect model of porcine.

## 2. Methods

### 2.1. Preparation of Dialdehyde Methyl Cellulose (DAC)

Methyl cellulose (Cat. M0512, Sigma-Aldrich, St. Louis, MO, USA) was dissolved in distilled water at room temperature, and then 0.05 M, 0.1 M, 0.2 M sodium periodate solution (NaIO_4_, Cat. 30323, Sigma-Aldrich, St. Louis, MO, USA) was added in for the process of oxidation reaction in dark as shown in Table 1. After 24 h, ethylene glycol (Cat. 324558, Sigma-Aldrich, St. Louis, MO, USA) was added into the mixture to react with residual NaIO_4_. The mixture solution was loaded into dialysis tubing (Spectrum™, MWCO 6–8 kD) and dialyzed for several days. Purified dialdehyde methyl cellulose (DAC) was obtained by lyophilization and stored at −20 °C until use. Aldehyde functional groups were characterized by FTIR, and the oxidation degree of DAC was quantified by KI methods [25].

### 2.2. Preparation of N-Succinyl Chitosan (SUC)

S-CS was synthesized according to Kacey G. Marra et al. with slightly modification [26]. Chitosan solution (Deacetylation > 75%, C3646, Sigma-Aldrich, St. Louis, MO, USA) was prepared by dissolved 0.25 g of chitosan in 20 mL of 5% *L*-lactic acid (Sigma-Aldrich, St. Louis, MO, USA) solution and 80 mL of methanol (Sigma-Aldrich, St. Louis, MO, USA). After gently stirring, 0.75 g of succinic anhydride (Sigma-Aldrich, St. Louis, MO, USA) was added in. After 24 h, succinyl modified chitosan (SUC) was precipitated by adjusting the pH value to 7. Precipitate was re-dissolved in distilled water and dialyzed (Spectrum™, MWCO 6-8 kD) for several days. Purified SUC was obtained by lyophilization stored at −20 °C until use. Functional groups were characterized by FTIR (PerkinElmer Spectrum 100, Waltham, MA, USA). Ninhydrin assay was further used for succinyl substitution rate determination [27] according to the following formula “succinylation % = ([NH_2_]_o_ − [NH_2_]_s_)/[NH_2_]_o_ × 100%”.

### 2.3. DAC-SCS Hydrogel Preparation

DAC and SUC were dissolved in PBS separately at a concentration of 70 mg/mL and 10 mg/mL. The crosslinked DAC-SUC hydrogels were formed by mixing DAC and SUC solutions at 1:1 and 1:3 volume ratios at room temperature. Table 2 summarized the parameters of the DAC-SUC hydrogel evaluated in the study.

### 2.4. Rheological Properties of DAC-SCS Hydrogel

The visco-elastic behavior of the hydrogels was monitored by Physica MCR Rheometer (MCR501) by oscillation frequency sweep model with a controlled strain of γ = 0.01 rad. The hydrogel was pre-cured on the parallel plate at 37 °C for 30 min before testing, and the range of the frequency was from 1 to 100 rad s^−1^. The values of complex shear modulus |G*|, storage modulus G’, loss modulus G” and the phase shift angle δ, were plotted as a function of frequency.

### 2.5. Mass Remaining of DAC-SUC Hydrogel

Mass remaining studies were conducted on hydrogel in phosphate buffered saline (PBS, pH 7.4, Cat. 10010023, Life Technologies Corporation, Grand Island, NY, USA) under 37 °C, 5% CO_2_. In brief, 1.1 mL of liquid-state hydrogel solution were introduced into the mold and allowed to set for 30 min to form a gel. After transferring hydrogel into a tube, 10 mL of PBS was added to each well. At a specific time point, the hydrogel was removed, blotted gently with filter paper to remove surface water, and the swollen hydrogel was weighed (W_s_) and recorded. The mass remaining (%) was calculated using the formula W_s_/W_i_ × 100%, where W_i_ is the initial weight of hydrogel (W_i_) on day 0. All experiments were conducted in six repetitions.

### 2.6. Cell-Compatibility Evaluation of DAC-SCS Hydrogel

The cell-compatibility of DAC-SUC hydrogel was evaluated by extraction medium and direct cultured method and stained with LIVE/DEAD^®^ reagent. The extraction medium was prepared by incubating the hydrogel with standard culture medium at a 1:10 (hydrogels: medium) extraction ratio for 72 h at 37 °C. The standard culture medium and medium containing 0.1% Triton X-100^®^ was used as control (Ctrl) and negative (NC) control groups. Then using the extraction medium for chondorcyte cultivation. To quantified cell viability, WST-1 and LDH assay were used for cell-compatibility test according to the kits’ instruction manual.

### 2.7. Bone Marrow-Derived Mesenchymal Stem Cell (BM-pMSCs) Culture

Briefly, the mesenchymal stem cells (BM-pMSCs) were obtained from the adult porcine iliac crest using the bone marrow aspiration technique. Bone marrow was obtained by 11-gauge needle attached to a heparinized syringe using an Animal Care and Use Protocol approved by the Far Eastern Memorial Hospital IACUC. A volumes of 3 mL of bone marrow cells were collected in a centrifuge tube and added 5 mL of phosphate buffer salt (PBS) solution (pH 7.4) to mix well by inversion. Then 8 mL of the bone marrow-saline mixture was layered onto the 3 mL Ficoll-Paque (Cat. Cytiva 17-1440-02, Sigma-Aldrich, St. Louis, MO, USA) and centrifuged at 600 g for 30 min at room temperature. After centrifugation, the upper layer was discarded, and the opaque interface carefully transferred to another clean tube. The BM-pMSCs were incubated in high-glucose DMEM medium (Cat. 12100, Gibco, Waltham, MA, USA) containing 10% FBS and 1% (100×) antibiotic-antimycotic solution at 37 °C in a humidified atmosphere containing 5% CO_2_. The medium was changed twice a week. BM-pMSCs were also characterized by flow cytometric analysis of CD11b, CD31, CD45, CD44, CD90 and CD105. Flow cytometric analysis was performed using a FACSCalibur flow cytometer. Cells were gated using forward and side scatters to exclude debris and cell aggregates.

### 2.8. MSC Tri-Linage Differentiation

For chondrogenesis, 4×10^5^ BM-pMSCs were centrifuged at 500g for 10 min, the medium was discarded and changed to chondrogenic differentiation medium after one day of cultivation. Chondrogenic differentiation medium is composed of DMEM-high glucose (Cat. 12100, Gibco) with ITS Premix (BD Biosciences, San Jose, CA, USA), 10^−3^ M sodium pyruvate, 1.7 × 10^−4^ M *l*-ascorbic acid-2-phosphate, 3.5 × 10^−4^ M proline, 10^−7^ M dexamethasone (Sigma-Aldrich, St. Louis, MO, USA), and 10 ng/mL of TGF-β1 (R&D Systems, Minneapolis, MN, USA). Medium was changed every 2–3 days. Chondrogenic induction was then further confirmed by Alcian blue staining.

For osteogenesis, BM-pMSCs were plated in six-well-plates at a density of 2 × 10^5^ cells. MSCs with nearly 90% confluence were exposed to the osteogenic differentiation medium for 14 days. Osteogenic differentiation medium contains DMEM-HG supplemented with 10% FBS, 10^−7^ M dexamethasone, and 25 μg/mL of *l*-ascorbic acid for 2 weeks. After induction, the osteogenic phenotype was assessed by staining with Alizarin red, which stains calcium-rich mineral deposits.

For adipogenic differentiation, BM-pMSCs were exposed to the adipogenic differentiation medium after 90% confluence. The adipogenic differentiation medium contains low-glucose DMEM supplemented 10% FBS, 1 mM of dexamethasone, 0.5 mM of 3-isobutyl-1-methylxanthine (IBMX), and 60 mM of indomethacin. After induction for 3 weeks, the cells were stained with oil red-O to evaluate the adipogenesis results.

### 2.9. Create Osteochondral Defect and Hydrogel Implantation

Six miniature pigs were used in this study. All operations and interventions were performed under general anesthesia. Osteochondral defect was created in medial condyles of both knee joints, while one received DAC-SUC hydrogel with 1 × 10^6^ BM-pMSCs and another remain defect only (spontaneous repair) or received hydrogel and covered with periosteum as a control group. The diameter of the defect was 6.5 mm with 5 mm depth. A pre-operative antibiotic was used. Zoletil^®^ (0.55–0.8 mg/kg body weight) was injected intramuscularly for pre-anesthesia, and then Citosol^®^ (1.11–1.66 mg/kg body weight) was slowly injected intravenously for deep anesthesia. Flunixin^®^ (1.0–2.2 mg/kg) was injected intramuscularly as analgesic. Porcines were allowed free movement after surgery and sacrificed after six months. Then GAG histological analysis and immunohistochemical analysis (Type II and X collagen) were performed for extracellular matrix evaluation.

### 2.10. Histological Procedures, Histological Score, and Statistics

Specimens from the knee joints were decalcified, embedded in paraffin, and cut into 5 μm slices. Fast Green/Safranin-O was used to visualize and compare the degree of both cartilage and bone repair obtained. Sections were immersed in 0.05% Fast Green in water for 5 min and quickly washed in 0.1% acetic acid, then immersed in 0.1% Safranin-O in water for 5 min and washed in ethanol. For immunohistochemical staining, sections were pretreated with xylene and re-hydrated with a graded ethanol-water series. Anti-collagen II antibody (Cat. no. AB746P, Millipore, Temecula, CA, USA) and anti-collagen X (Cosmo Bio, Carlsbad, CA, USA) staining were used, respectively. Two secondary antibodies were used including anti-rabbit (Cat. GR608H, Biocare Medical, Pacheco, CA, USA) and goat anti-mouse (Cat. GM601H, Biocare Medical, Pacheco, CA, USA). Hematoxylin was performed by incubating rehydrated sections for 2 min followed by 3 rinses with deionized water. The score published by modified O’Driscoll score [28] and Wakitani score [29] were used for sections evaluation of both knees.

### 2.11. Statistical Analysis

All the data were presented as mean  ±  SEM and analyzed using GraphPad Prism software (Version 6.0). *p* values were calculated by one-way ANOVA for multiple comparisons using the indicated post hoc Bonferroni test with at least 3 or more replicates and where two groups were compared, non-paired Student’s *t*-test was used; in vivo data was analyzed using two-way ANOVA.

## 3. Results

### 3.1. Characterization of DAC, SUC and the Hydrogel

**Figure 1:**
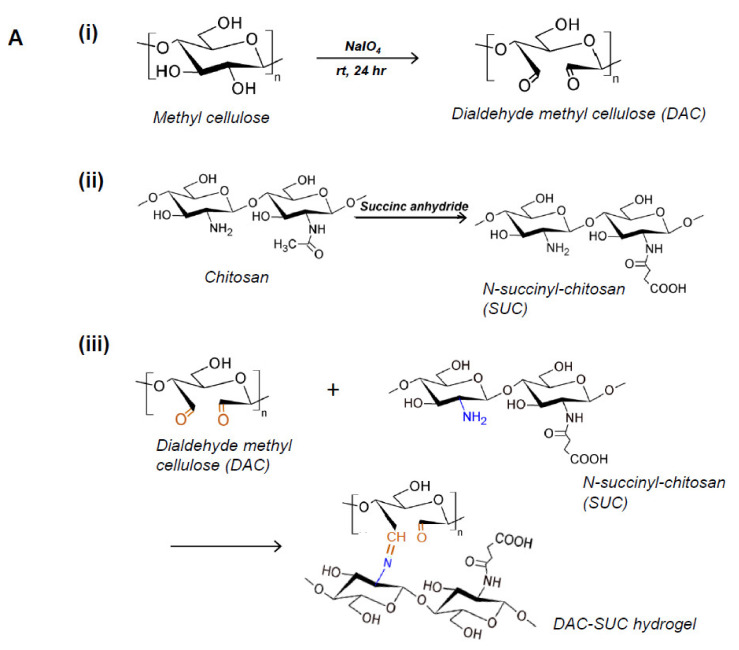

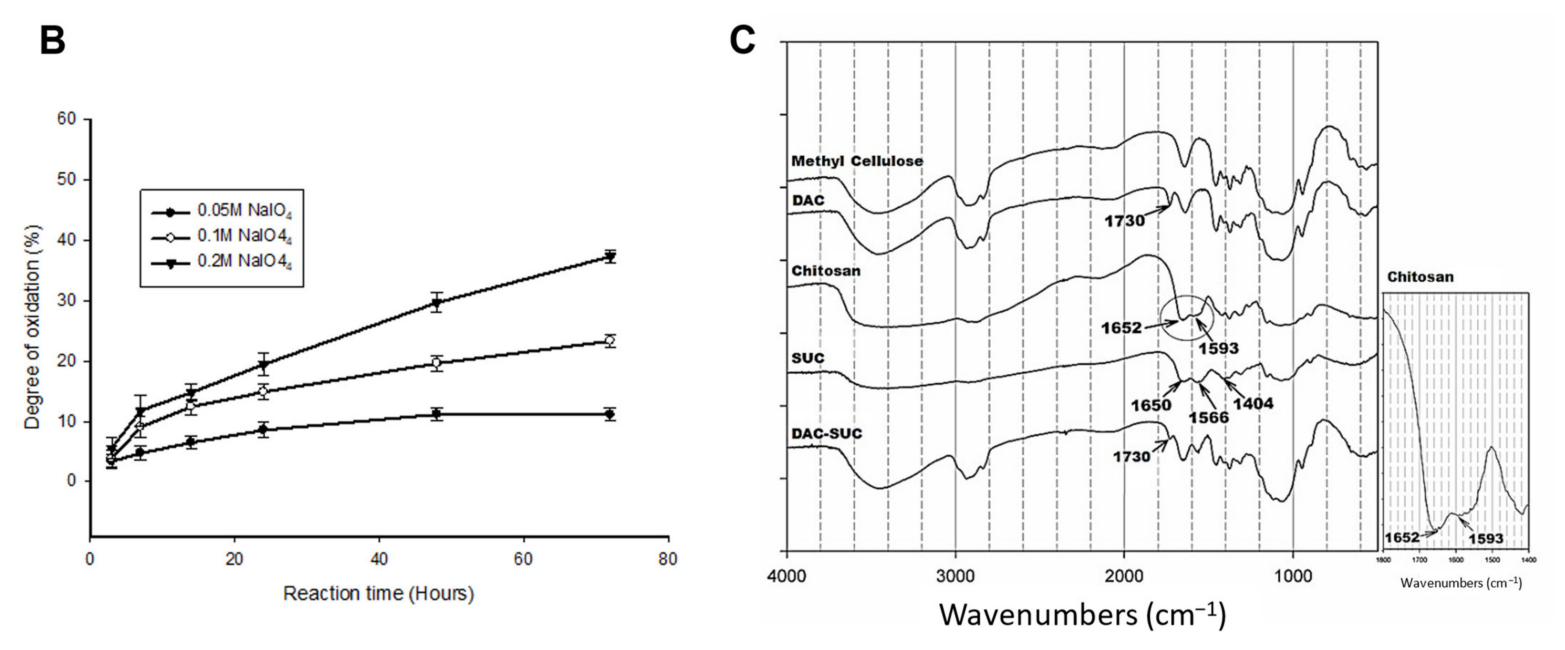
Characterization of DAC, SUC and the hydrogel. (**A**) Schematic representation of the synthetic route of (**i**) 2,3-dialdehydecellulose (DAC), (**ii**) succinyl-chitosan (SUC) and (**iii**) DAC-SUC hydrogel. (**B**) Oxidation percentage of DAC with different concentration of sodium periodate and reaction time. (**C**) Functional groups of methyl cellulose, DAC, chitosan, SUC, and DAC-SUC hydrogel identified by FT-IR spectra.

Methyl cellulose was oxidized by sodium periodate to create aldehyde functional groups (dialdehyde methyl cellulose; DAC), and succinyl-chitosan (SUC) was synthesized with succinic anhydride. Therefore, the in situ forming DAC-SUC hydrogel could be prepared by simply mixing DAC and SUC two solution together. Figure 1A showed the functional group created reactions and the hydrogel formed chemical reaction.

We evaluated the influence of sodium periodate concentration and reaction time, results showed that increase both two factors could lead to DAC oxidation degree increased, as shown in Figure 1B. In present study, 8.5%, 15%, and 20% oxidized cellulose was chosen for further investigation, and the degree of succinyl modified chitosan was 31.5% ([NH_2_]_o_ = 2.03 ± 0.084 μmole/mg; [NH_2_]_s_ = 1.39 ± 0.043 μmole/mg). Functional groups of methyl cellulose, DAC, chitosan, SUC, and DAC-SUC hydrogels were also qualified by Fourier transform infrared (FTIR) spectroscopy, as shown in Figure 1C. Comparing with methyl cellulose and DAC, the spectrum of DAC shows a new absorption peak around 1730 cm^−^^1^, which related to the aldehyde functional groups. Two basic characteristic peaks of the chitosan 1652 cm^−1^ (NH_2_ deformation) and 1593 cm^−1^ (N–H bend) as reported in the literature [30]. After succinylation, the peak of SUC was a little bit change, two absorption peaks were appear at 1650 cm^−1^ and 1404 cm^−1^ corresponded to the formation of –CO–NH–, and the absorption peaks at 1568 cm^−1^ is attributed to the N–H absorption [19]. After mixing DAC and SUC solution together, we found the absorption peak at 1730 cm^−1^ was smaller compared with that of DAC because of the crosslinking reaction between C=O of DAC and NH_2_ of SUC.

### 3.2. Rheological Properties and Mass Remaining of the Hydrogel

**Figure 2:**
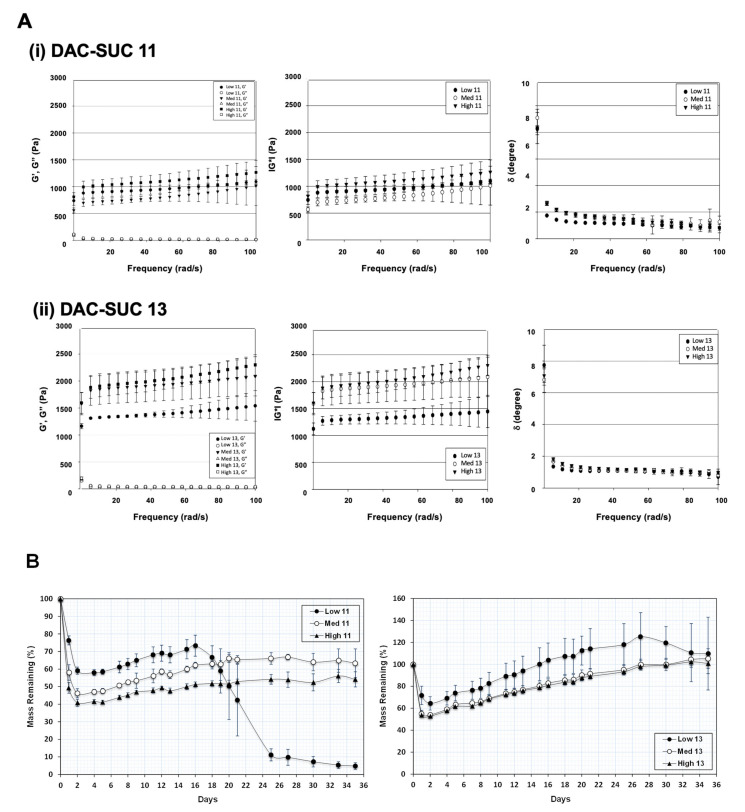
Rheological properties and mass remaining of the DAC-SUC hydrogel. (**A**) Rheological characterization of (**i**) DAC-SUC 11 and (**ii**) DAC-SUC 13 hydrogel (frequency sweep) shown as the storage modulus (G’), loss modulus (G”), complex modulus |G*| and tangent of the phase angle (δ). The storage modulus G′ was increased with oxidation rate of DAC increase. (**B**) Mass remaining of DAC-SUC hydrogels. The weight loss of hydrogel was recorded for 35 days and DAC-SUC13 hydrogel could exist over 35 days.

Figure 2A showed the rheological properties of soft hydrogel, (i) indicated the results of DAC and SUC mixing volume ratio 1:1, and (ii) indicated the results of DAC and SUC mixing volume ratio 1:3. Storage modulus G′ represents the elastic part and loss modulus G′′ represents the viscous part of the hydrogel. Results revealed that the storage modulus (G′) was higher than the loss modulus (G′′), indicated that the hydrogel showed more elastic-like behavior. Additionally, the storage modulus G′ was increased with oxidation rate of DAC increase (High DAC-SUC > Medium DAC-SUC > low DAC-SUC). Complex shear modulus |G*| could represent the stiffness of a hydrogel. Results showed that the |G*| was increased with SUC concentration increased. The value of |G*| in DAC-SUC 11 groups were among 0.5 kPa to 1.5 kPa, and the value in DAC-SUC 13 groups was increased from 1 kPa to 2.5 kPa. The increase |G*| may be related to the increase of C=N crosslinking reaction between DAC and SUC polymers in the hydrogel.

The ratio of G” and G’ is related to tangent of the phase shift angle δ according to the formula tan δ = G”/G’. Phase shift angle δ was directly obtained from Physica MCR Rheometer (MCR501) software. δ would be equal to 0° for purely elastic material, while it would be equal to 90° for purely viscous material. Viscoelastic materials would have both properties and possess a δ value between 0 and 90°. For DAC-SUC hydrogel, δ values were lower than 3 indicating these hydrogels showed more elastic-like behavior.

Of note, the residual weight of hydrogels was observed for 35 days and only low DAC-SUC13 hydrogel had the least degradation, as shown in Figure 2B. The residual weight of hydrogels was decreased with SUC concentration decreased (DAC-SUC 11 < DAC-SUC 13). Low DAC-SUC 11 hydrogel was gradually degraded after two weeks and totally degraded at week 5. For Medium DAC-SUC 11 and High DAC-SUC 11 hydrogels, the residual wet weight were about half of the initial wet weight.

### 3.3. Cell-Compatibility Evaluation of DAC-SCS Hydrogel

**Figure 3:**
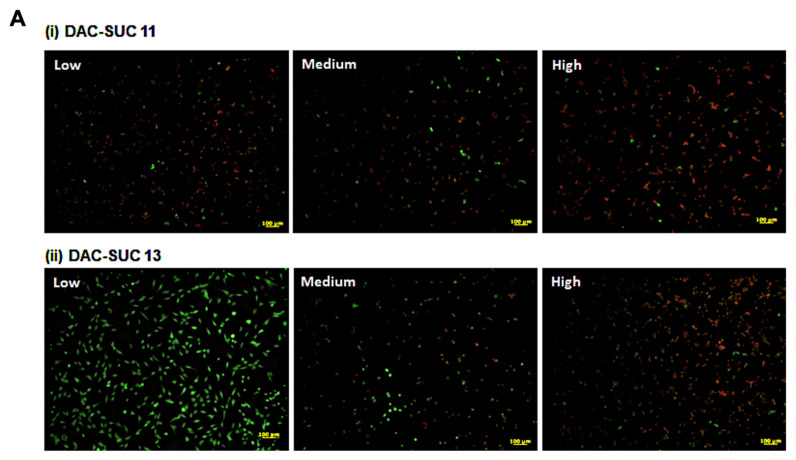

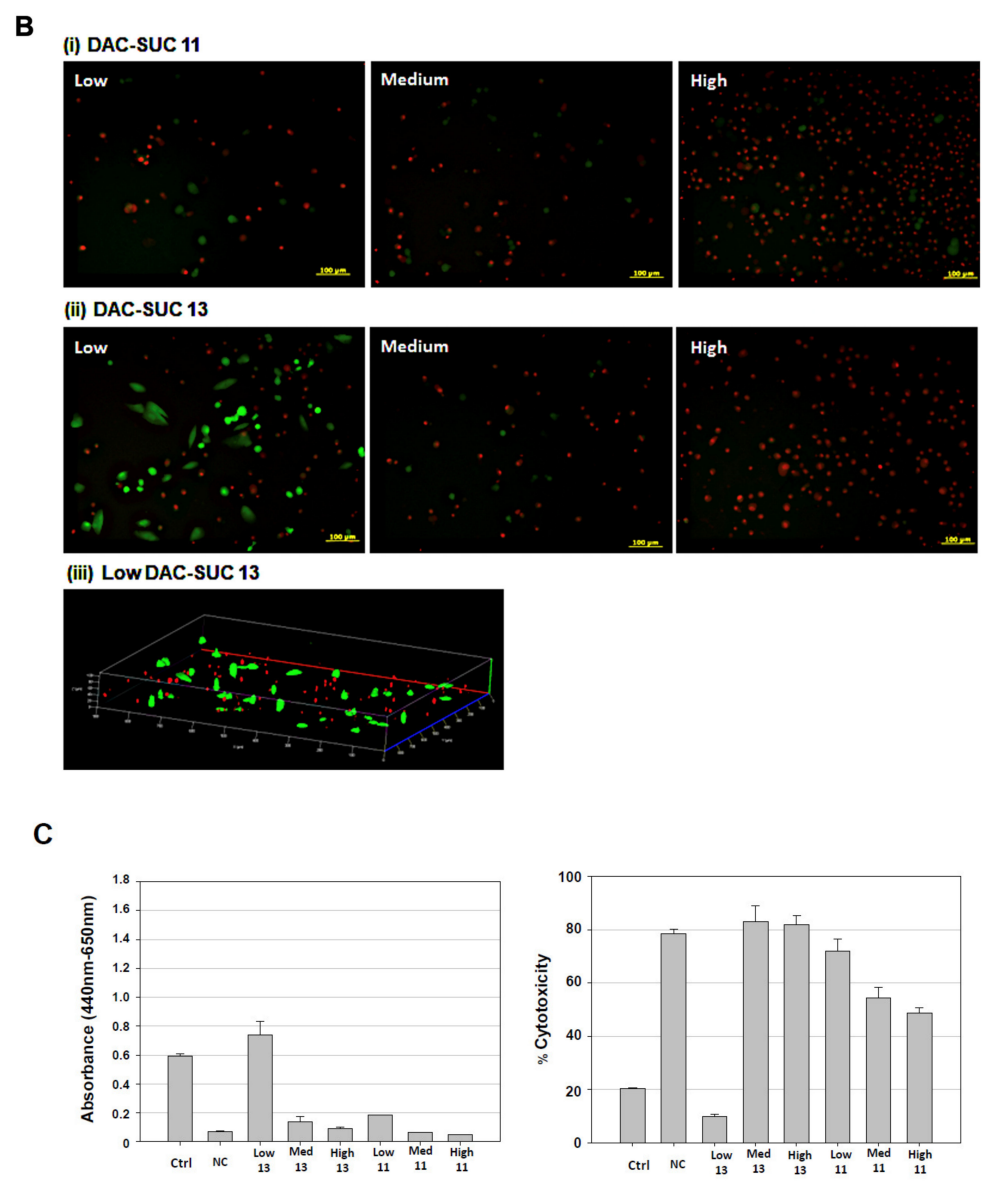

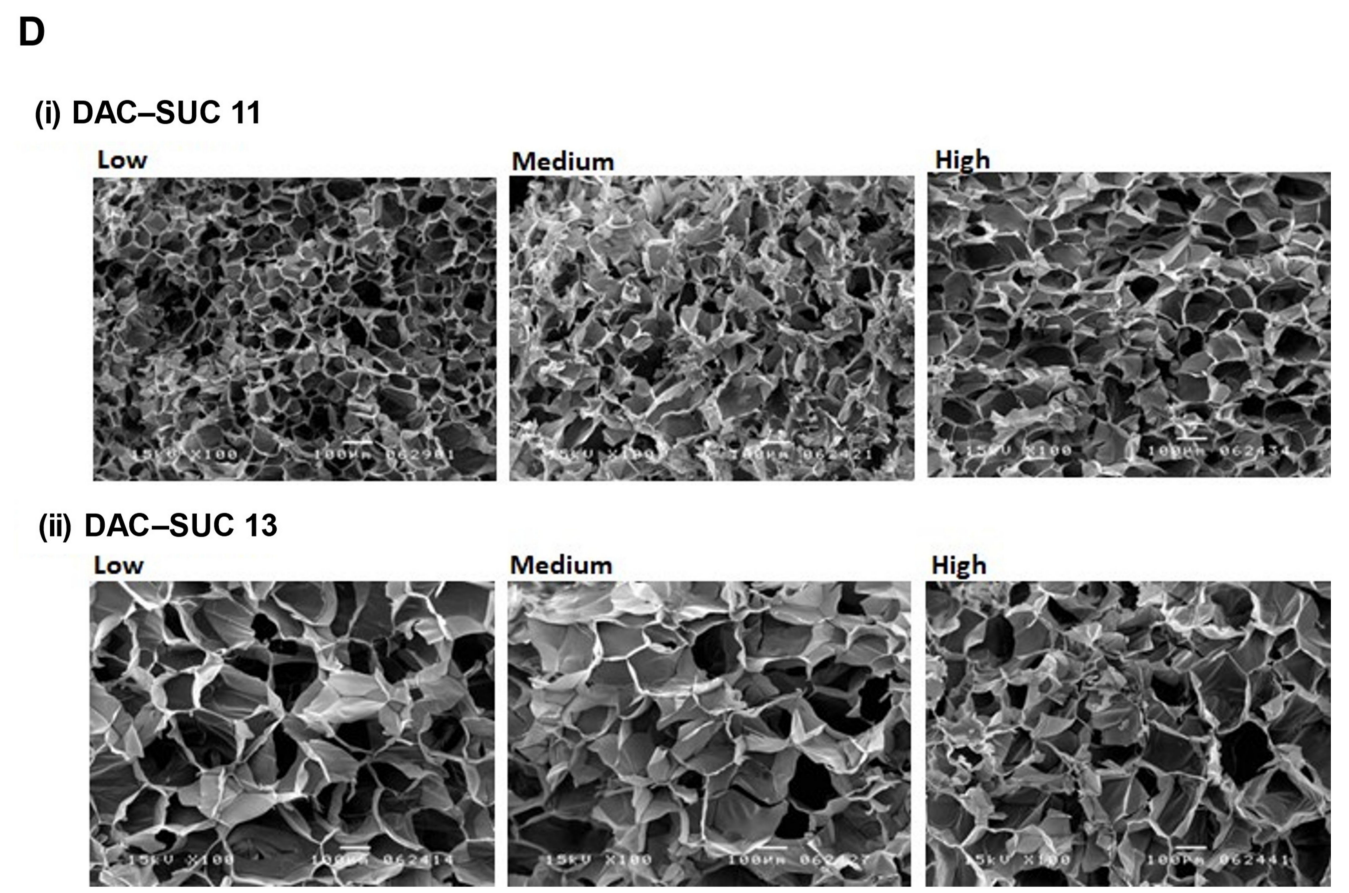
Cell-compatibility of DAC-SUC hydrogel. (**A**) Cells were treated with (**i**) DAC-SUC11 or (**ii**) DAC-SUC13 hydrogel extraction medium and then stained with LIVE/DEAD^®^ reagent. Most cells were shown green spots (alive) in Low DAC-SUC13 group. (**B**) Cells were encapsulated in (**i**) DAC-SUC11 or (**ii**) DAC-SUC13 hydrogels and then stained with LIVE/DEAD^®^ reagent. Most cells were shown green spots (alive) in Low DAC-SUC13 group. (**iii**) The 3D image of Low DAC-SUC13 hydrogel. (**C**) Cell viability ad cell death were evaluated. Results showed that cells were alive well in Low DAC-SUC13 hydrogel group. (**D**) SEM images of hydrogels. The hydrogels displayed a continuous and porous structure.

When cell cultured with DAC-SUC13 hydrogel extraction medium, most cells were alive and shown green fluorescence. However, with the oxidation degree of DAC increased, the number of green spots were decreased. For DAC-SUC11 hydrogel groups, only very few green spots can be found, as shown in Figure 3A. Figure 3B showed the LIVE/DEAD^®^ staining images of cell encapsulated in the hydrogel, only DAC-SUC13 hydrogel present positive results, most of the cells were alive in the DAC-SUC13 hydrogel. To further confirm the image under 3D hydrogel, we used confocal to scan layer by layer and reconstruct Low DAC-SUC13 hydrogel images. Results showed that most of the cells were present green spots, and the cell distributed well.

The cell viability and cytotoxicity could be quantified by WST-1 and LDH assay, as shown in Figure 3C. Results revealed that Low DAC-SUC13 hydrogel sustained cell survival and possess very low toxicity. These observations showed that Low DAC-SUC13 hydrogel without add any other crosslinking agents could maintain cell viability and has the potential to be used as in situ injectable scaffolds for cartilage tissue engineering.

Figure 3D showed cross-section SEM images of DAC-SUC hydrogel, and it characterized the microstructure morphologies. The hydrogels displayed a continuous and porous structure. The pore size of DAC-SUC13 hydrogels were larger than that of DAC-SUC11.

### 3.4. MSC Characterization and Tri-Differentiation Potential

**Figure 4:**
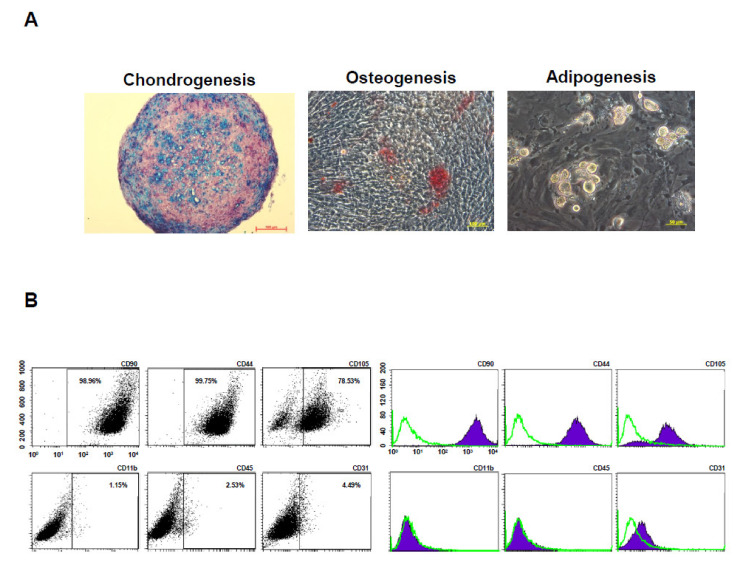
Characterization of pMSCs by tri-lineage differentiation and surface markers. (**A**) Representative images of in vitro tri-lineage differentiation potential of porcine bone marrow MSC (BM-pMSC) toward chondrogenic, osteogenic, and adipogenic lineages confirmed by histochemical staining with Alizarin red, Oil red O, and Alcian blue staining, respectively. BM-pMSCs exhibited multi-differentiation capacity of adipogenesis, osteogenesis, and chondrogenesis. (**B**) Representative results of flow cytometric analysis for cell surface markers using freshly isolated BM-pMSC from porcine. The population is positive for previously described MSC markers (CD90, CD44, and CD105) and negative for CD11b, CD45 and CD31 expressions from passages p5.

To evaluate multilineage differentiation potential, the BM-pMSCs were induced to differentiate towards chondrogenic, osteogenic, and adipogenic induction. The results demonstrated that BM-pMSCs are able to differented into chondrogenic, osteogenic, and adipogenic lineages, as observed in Figure 4A. In order to determine the phenotypic marker profiles of stem cells, the BM-pMSCs were stained and showed positive expression of classical MSC surface markers CD44, CD90, and CD105, and negative expression of CD11b, CD31, and CD45 by flow cytometry (Figure 4B). These data confirmed that BM-pMSCs had the general properties of MSC in surface markers and differentiation potentials.

### 3.5. BM-pMSC Improves Cartilage Repair in Porcine

**Figure 5:**
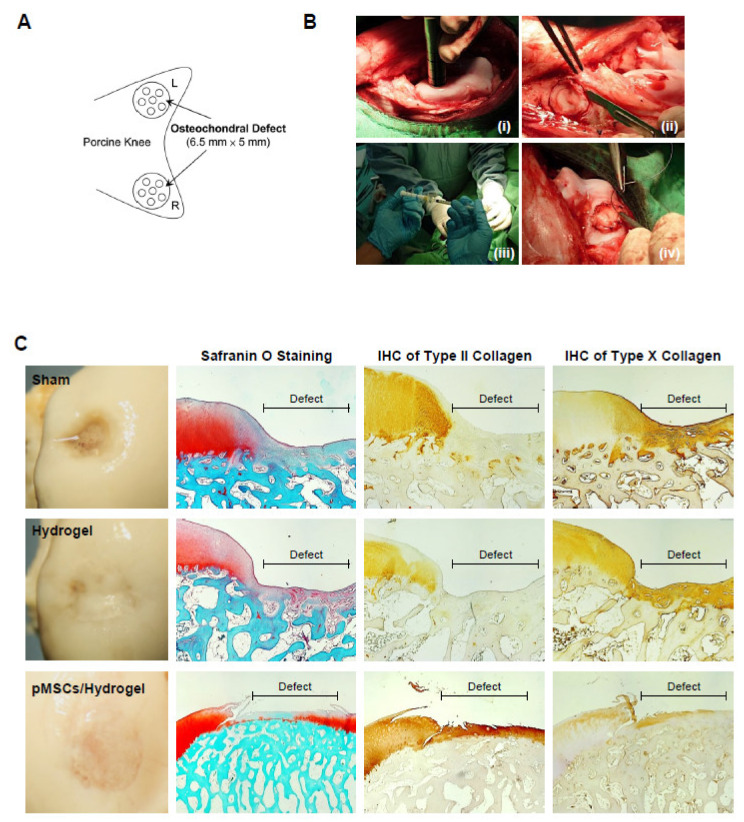
pMSC-hydrogel-implants enhance cartilage regeneration in the large-animal OCD model. (**A**) The OCD animal model in porcine. (**B**) The surgical procedure of the OCD regeneration in porcine. (**i**) The OCD were generated using an electric drill in femoral patellar groove; (**ii**) a 6.5 mm × 5 mm area of OCD was obtained; (**iii**) the pMSC-hydrogel-scaffold was implanted into OCD; and (**iv**) the patella was relocated and sutured the wound in layers. (**C**) Microscopic appearance and histological stained sections of the repaired sites in situ gelation at 6 months post-implantation. Macroscopic view of operated femoral condyle with pMSC in hydrogel implants. After paraffin-embedded and microsection, staining the sections by Safranin O, type II collagen and type X collagen.

For identifying the function of cartilage repair of BM-pMSC-based hydrogel scaffolds, we generated a 6.5 mm in diameter and 5 mm in depth osteochondral defect of either knee joint in porcine (Figure 5A). The BM-pMSC/DAC-SUC hydrogel were injected after irrigating the joint with sterile isotonic saline and covered with periosteum, as shown in Figure 5B. After 6 months, defect site in BM-pMSC/DAC-SUC hydrogel group was filled with reddish-white tissue distinct from the native articular cartilage. The periphery of the defect was partially filled with white tissue which is relatively smoother. During the observation period, the defect sites in the sham group were irregular and depressed. The border areas of the defect sites were distinct compared to the near-native articular cartilages (Figure 5C). Next, to quantify the whole cartilage regeneration by BM-pMSC/DAC-SUC hydrogel, we used the preclinical cartilage repair scoring systems, modified O’Driscoll score and Wakitani score. The assessments were commonly used in the evaluation of the extent of cartilage damage or the success of cartilage regeneration by macroscopic observation and histological analysis. Proteoglycan content of Safranin O staining was significantly enhanced in regenerated cartilage of BM-pMSC/DAC-SUC hydrogel implantation group. The modified Wakitani score of the regeneration of cartilage and IHC staining of type II collagen in cartilage defect after implanting BM-pMSC/DAC-SUC hydrogel scaffold was increased compared with sham/DAC-SUC hydrogel group. A hypertrophy-associated type X collagen was also suppressed by BM-pMSC/DAC-SUC hydrogel implants. In addition, a trend towards an increased O’Driscoll histology score was significantly noted in the MSC-based hydrogel-scaffold group compared to the sham or hydrogel group (Table 3).

**Figure 6:**
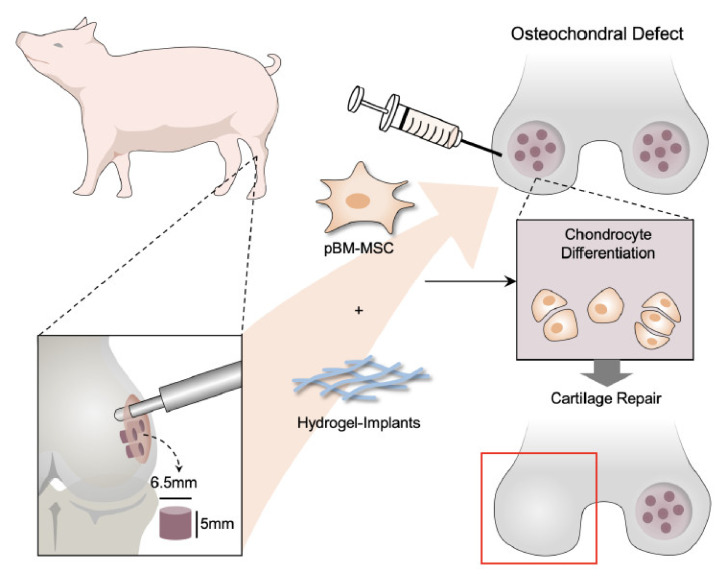
Action of pMSC-based therapies in cartilage regeneration in porcine OCD model. Schematic illustrations of the tissue engineering approach for cartilage repair. In this study, pMSCs were applied as cell replacement therapy because of its chondrogenic differentiation potential. Cell-compatible DAC-SUC hydrogel could encapsulated pMSC well improved the limitation of clinical treatment such as donor site mobility, secondary surgery, and implanted chondrocyte loss. Additionally, the formation of new cartilage by pMSCs and the deposition of ECM synthesis and type II collagen can also be stimulated by combining pMSCs and 3D DAC-SUC hydrogel.

For treating cartilage defects, the microfracture requires drilling into the subchondral bone to release bone marrow that forms an MSC-rich clot at the site of the wound. We created tiny cracks in the subchondral bone to allow blood and cells from the bone marrow to infiltrate the injury site and compared them to the sham group. The results revealed that the full-thickness cartilage defect was filled with overgrown fibrous cartilage-like cells. The deeper zones of the microfracture holes had undergone mature subchondral bone remodeling, with evidence of the hyaline-like cartilage in the superficial zone was positive for Safranin O staining and type II collagen expressions (Figure S1). In contrast, in the sham group, the sham group showed some scar tissue formation. Of note, expression of type X collagen was not suppressed. Hence, the cartilage repairing scores indicated that MSC based hydrogel-scaffold implant is higher than the microfracture group. The above observations suggest that combination of MSC and hydrogel-scaffold implant is sufficient to be used in tissue engineering for cartilage regeneration in the large-animal model (Figure 6).

## 4. Discussion

At articular surfaces in the porcine OCD animal model, MSC implants may produce fibrocartilage instead of the normal hyaline cartilage. It is usually derived from bone marrow-stimulating cartilage repair techniques such as microfracture and may only produce short-term repair, long-term durability was inadequate. As the native hyaline cartilage was repaired with fibrocartilage consisting of primarily type I collagen, which is prone to rapid degeneration and less adaptable than its hyaline counterpart [31]. Microfracture alone to access the autologous MSC populations does not recruit enough reparative cells and growth factors to promote adequate native cartilage repair [32]. The repair tissue did not resemble normal hyaline cartilage, and the implants exhibited varying degrees of integration with the surrounding region. However, these treatments still do not have the full regeneration of cartilage into the native form.

ACI is another well-known procedure for cartilage defect repair; however, it possesses donor site mobility, secondary surgery, and implanted chondrocyte loss limitations. Thus, several different kinds of scaffolds been developed. Svensson et al.’s efforts [14] had reported the response of chondrocytes on native and chemically modified bacterial cellulose (BC). Their results indicated that the BC scaffold supported chondrocytes proliferation and enhanced collagen type II synthesis. Ke Wang et al.’s study revealed that BC and silk fibroin (SF) double-network hydrogel possess high mechanical strength and biocompatibility, but they did not show any results related to matrix synthesis [15]. Another highly porous membranes prepared by 2,3-dialdehydecellulose (DAC) as a scaffold in tissue engineering has been investigated by Roy Chowdhury’s group [16]. DAC can be successfully prepared from cellulose or methylcellulose by using sodium periodate oxidation process and possess well biodegradation and biocompatibility properties. Recently, a scaffold prepared by entrapped carboxymethyl cellulose in a poly(vinyl) alcohol network has been introduced, but the scaffold needed further glutaraldehyde crosslinked process [33]. Researchers also tried to use an electrospinning technique to prepare a hyaluronic acid/poly (3-hydroxybutyrate)/chitosan/carbon nanotubes scaffold for cartilage repair, the scaffold has enough tensile strength, and the hydrophilic ability of the scaffold could be enhanced by the increase in hyaluronic acid concentration [17]. However, using scaffolds for cartilage repair have some disadvantages, such as inconvenient to use, defect site incompletely filling and cell loss. Orthopedics need to trim the scaffolds before implantation. Additionally, the seeded cell within the scaffold would be gradually loss due to large porous structure, thus influence the repair ability of cells in vivo.

In the present study, we successfully developed a method for in situ forming DAC-SUC hydrogel preparation. Dialdehyde functional groups on methyl cellulose was created by sodium periodate, increase the oxidant concentration and reaction time would enhance DAC oxidation degree. The FTIR peaks of aldehyde functional groups could be found around 1730 cm^−1^. After mixing DAC with succinyl modified chitosan (SUC) together, the peak of the aldehyde functional groups was decreased, and results were similar to another research [25]. According to rheological evaluation, it showed that the DAC-SUC hydrogel was an elastic-like hydrogel because of high storage modulus (G′), and the storage modulus G′ was increased with oxidation rate of DAC increase. Additionally, the complex shear modulus |G*| of the hydrogel was increased with SUC concentration, as well as the |G*| value in DAC-SUC 13 groups, by about 1kPa–2.5kPa. The increase in |G*| may be related to the increase in the C=N crosslinking reaction between DAC and SUC polymers in the hydrogel, which led to a stiffer hydrogel formation. These hydrogels could assist cell for matrix production over 35 days. The possible degradation mechanism of the hydrogel would be hydrolysis. However, we found that most of the cells cultured with DAC-SUC11 hydrogel extraction medium or within the hydrogel were dead. This phenomenon might be due to the toxicity of aldehyde functional groups on DAC, these aldehyde functional groups may react with cells, and further lead to cell death. The optimal DAC-SUC mixing ratio is 1 to 3 for form in situ forming hydrogel.

BMSCs have become an ideal selection for the regeneration of cartilage, and the cells grow and multiply rapidly and we obtained a significant number of cells at the initial passages. Recent in vitro studies have demonstrated the promotion of chondrogenesis of hMSCs encapsulated in hydrogels [34,35]. However, utilizing the functional properties of the articular cartilage regeneration using only BMSCs remains challenge. Considering the great potentials of BM-MSCs in cartilage repair, we applied the BM-pMSCs into DAC-SUC hydrogels. In this study, we found that pMSC can induce chondrogenesis in vitro, and DAC-SUC hydrogel can function as a stem cell carrier to promote cartilage repair in the OCD of either knee joint in porcine. Using a porcine model, we found that DAC-SUC hydrogel carrying BM-pMSC enhanced the repair of cartilage defects after 6 months. The presence of glycosaminoglycans in the extracellular matrix was determined using Safranine-O staining and immunohistochemical staining of healed tissue for collagen types II provided definitive evidence of repair. Since collagen type II mainly exists in hyaline cartilage, the expression of collagen type II is increased. It is worth noting that DAC-SUC hydrogel scaffold was superior to microfracture in terms of inducing repairing cartilage defects. Type X is the collagen present in the calcified cartilage in contact with the subchondral bone and absent in chondrogenesis [36]. Our findings indicated that type X collagen was reduced in DAC-SUC hydrogel scaffold. Implantation of BM-pMSCs in DAC-SUC hydrogel scaffold into the OCD was later found to result in the suppression of type X collagen, implicating that the pMSCs-based hydrogel scaffold induced chondrogenic differentiation and contribute to the repair of articular cartilage.

Taken together, our results suggest that the applications of DAC-SUC hydrogel have a significant influence on the chondrogenic differentiation of BM-pMSCs, and that allowing for chondrogenic induction produced a regenerative tissue that reacted more positively to cartilage repair. Regardless, the hydrogel possesses several advantages, such as easy-to-use, complete defect filling, good cell compatibility as well as degradability. To our knowledge, this is the first study to demonstrate the beneficial effect of pMSCs-based hydrogel in cartilage repair in a porcine OCD animal model. Our findings also give new insight into the important implications to post-operation following clinical cell-based hydrogel treatment for cartilage regeneration. Future studies are required to further elucidate the specific underlying mechanism involving the role mechanical stimulation of chondrogenic differentiation of MSCs cultured in hydrogel.

## 5. The Limitations of BM-MSCs/DAC-SUC Hydrogel for Clinical Use

This study showed the application of BM-pMSCs encapsulated in DAC-SUC hydrogel for cartilage defects regeneration. Our findings suggest that BM-pMSCs in DAC-SUC hydrogel composite can treat articular cartilage defects. Implementing BM-pMSCs on DAC-SUC hydrogel microcarrier into a chondral defect porcine model could illustrate better chondrogenic differentiation than implanting MSC only. In addition, using this approach is more convenient because the resulting cell/hydrogel constructs could be used as a scaffold for direct transplantation, and the hydrogel will fill in defects completely. However, there are some limitations of DAC-SUC hydrogel for future clinical use. First, the degradation behavior of the hydrogel in vivo should be clarified by crosslinking fluorescence protein on DAC and SUC polymer and tracing the fluorescence signal to understand how the hydrogel degraded in vivo. Second, the cartilage regeneration signal pathway of BM-pMSCs in DAC-SUC hydrogel should be identified by real-time PCR, and evaluated by various regulators of chondrogenic differentiation, such as COLI, COLII, COLX, SOX-9, TGF-b1, and FGF-2 [37]. Third, the amplification of BM-MSCs ex vivo for mass production is limited. Regardless, BM-MSCs possess some disadvantages such as invasive pain, replicative senescence, and individual diversity [38,39]. Different kinds of stem cells can be encapsulated in the hydrogel and compared the regeneration results in future experiments, such as using adipose-derived stem cells from discard fat tissue and infrapatellar fat pad mesenchymal stem cells from discard knee fat pad tissue. Some reference indicated that infrapatellar fat pad mesenchymal stem cells possess an greater chondrogenic potential [40].

## 6. Conclusions

We demonstrated a method to prepare DAC-SUC hydrogel and found the optimal mixing ratio. The preparation of DAC-SUC13 hydrogel is very easy by simply mixing DAC and SUC solution together. The hydrogel can fill in defects completely, and cells or drugs can be encapsulated inside for further treatment. The DAC-SUC13 hydrogel had good cell compatibility as well as degradability. Most cells cultured in the hydrogel were keep alive, and the hydrogel could exist more than 35 days. Due to the 3D environment of the hydrogel, encapsulated pMSC can synthesize more glycosaminoglycans and type II collagen compared with clinical microfracture treatment. Importantly, the degeneration marker, collagen type X, was not found in new forming cartilage tissue in porcine OCD model. Such kinds of hydrogel can contribute to the development of future strategies in cartilage tissue engineering using stem cell for tissue repair or regeneration. The DAC-SUC13 hydrogel combined with stem cell is a potential treatment for cartilage defects repair in the future.

## Figures and Tables

**Table 1:** DAC preparation parameters.

Groups	Oxidation Parameters
Low DAC	0.05 M NaIO_4_ reacted with 7% Methyl cellulose for 24 h
Medium DAC	0.1 M NaIO_4_ reacted with 7% Methyl cellulose for 24 h
High DAC	0.2 M NaIO_4_ reacted with 7% Methyl cellulose for 24 h

**Table 2:** DAC-SUC hydrogel preparation parameters.

Groups	DAC Conc.	SUC Conc.	DAC: SUC (*v*/*v*)
Low DAC-SUC 11	70 mg/mL	10 mg/mL	1:1
Medium DAC-SUC 11	70 mg/mL	10 mg/mL	1:1
High DAC-SUC 11	70 mg/mL	10 mg/mL	1:1
Low DAC-SUC 13	70 mg/mL	10 mg/mL	1:3
Medium DAC-SUC 13	70 mg/mL	10 mg/mL	1:3
High DAC-SUC 13	70 mg/mL	10 mg/mL	1:3

**Table 3:** The preclinical cartilage repair scoring systems.

Groups	Sham	Hydrogel	pMSC/ Hydrogel	Microfracture
Modified O’Driscoll Score				
Surface regularity	0	1 (0–2)	1.5 (0–2)	2 (0–3)
Structural integrity	0	1 (0–2)	1.5 (0–2)	1 (0–1)
Safranin-O staining of the matrix	1 (0–1)	1 (0–3)	2 (1–2)	2 (1–3)
Thickness	0 (0–1)	1 (1–2)	1 (0–2)	0 (0–2)
Bonding to the adjacent cartilage	0 (0–1)	1	1 (0–2)	1 (0–2)
Cellular morphology	0 (0–2)	0.5 (0–2)	1 (0–4)	1 (0–4)
Hypocellularity	0 (0–1)	1 (2–3)	2 (0–3)	1 (1–3)
Chondrocyte clustering	1.5 (1–2)	1 (1–2)	1 (0–2)	1 (1–2)
Freedom from degenerative changes in Adjacent cartilage	1 (0–1)	1 (2–3)	2 (1–2)	1 (0–2)
Total Score (Scale Range 0–24)	3.5 (2–8)	8.5 (7–21)	13 (3–18)	10 (7–19)
Wakitani Score				
Surface regularity	3	2 (1–3)	2 (1–3)	1 (1–3)
Matrix staining	1 (2–3)	1 (0–3)	2 (1–2)	1 (1–2)
Thickness of cartilage	0 (0–2)	0	1 (0–2)	1 (0–2)
Integration of donor with host Adjacent cartilage	2 (1–2)	1 (0–1)	1.5 (0–2)	1 (0–2)
Cell morphology	1 (2–4)	2 (1–3)	1.5 (1–2)	2 (0–2)
Total Score (Scale Range 0–14)	7 (4–11)	6 (2–9)	8 (2–14)	6 (3–9)

## Data Availability

All data will be available upon request.

## Supplementary Materials

The following supporting information can be downloaded at: https://www.mdpi.com/article/10.3390/polym14071474/s1, Figure S1: Representative histological sections of tissue formation after 6 months with microfracture in the OCD animal model. Sections were stained with Safranin O in the sham and microfracture group. Microscopic appearance of the microfracture group showed thick regions of hyaline-like cartilage with some fibrocartilage and fibrous tissue in the chondral region. The subchondral region is composed of transitional and fibrous tissue. Immunohistochemistry analysis of representative chondrogenic markers, type II collagen and type X collagen, were stained.
