# Supplementary material for: Delivery of Mesenchymal Stem Cell in Dialdehyde Methylcellulose-Succinyl-Chitosan Hydrogel Promotes Chondrogenesis in a Porcine Model
Source: Polymers (Basel). 2022 Apr 5;14(7):1474. doi: 10.3390/polym14071474 (PMC9002496; doi:10.3390/polym14071474)
Supplement: Supplementary file 1 [file polymers-14-01474-s001.zip › polymers-1628556-supplementary.pdf]

polymers

MDPI

Supplementary Materials

Delivery of Mesenchymal Stem Cell in Dialdehyde Methylcellulose-Succinyl-Chitosan Hydrogel Promotes Chondrogenesis in a Porcine Model

Yu-Chun Chen [1,†], Hsiu-Jung Liao [2,†], Yuan-Ming Hsu [2], Yi-Shan Shen [2,3] and Chih-Hung Chang [2,4,*]

[1] Department of Chemical Engineering, National United University, Miaoli 360302, Taiwan; f94548002@gmail.com
[2] Department of Orthopedic Surgery, Far Eastern Memorial Hospital, New Taipei City 220216, Taiwan; liaohsiujung@gmail.com (H.-J.L.); femhortho@gmail.com (Y.-M.H.); katrina60943@gmail.com (Y.-S.S.)
[3] Department of Biomedical Engineering, National Taiwan University, Taipei 10617, Taiwan
[4] Graduate School of Biotechnology and Bioengineering, Yuan Ze University, Taoyuan City 320315, Taiwan
* Correspondence: orthocch@icloud.com
† These authors contributed equally to this work.

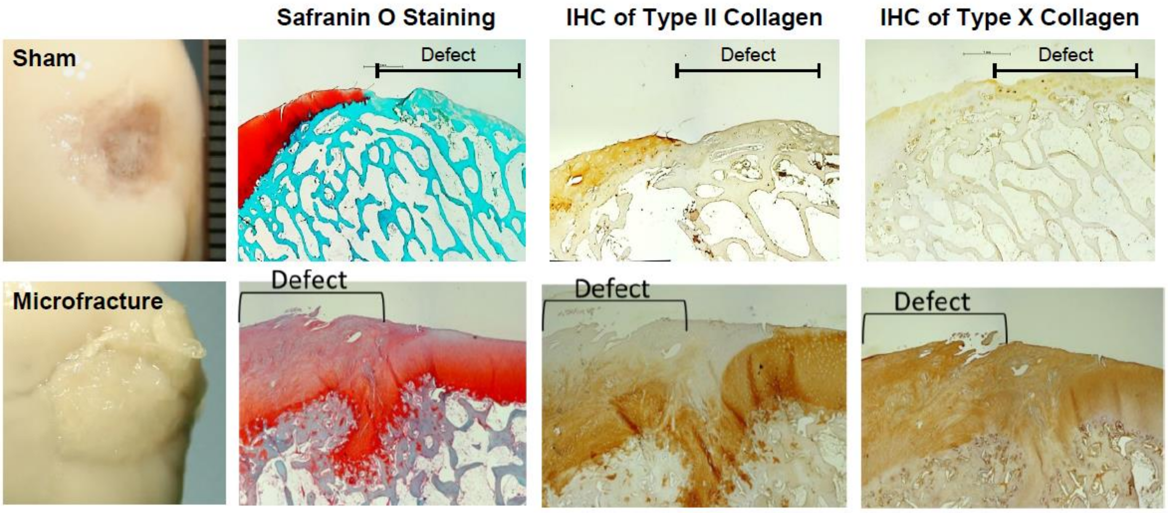

Figure S1. Representative histological sections of tissue formation after 6 months with microfracture in the OCD animal model. Sections were stained with Safranin O in the sham and microfracture group. Microscopic appearance of the microfracture group showed thick regions of hyaline-like cartilage with some fibrocartilage and fibrous tissue in the chondral region. The subchondral region is composed of transitional and fibrous tissue. Immunohistochemistry analysis of representative chondrogenic markers, type II collagen and type X collagen, were stained.
